# Total and Phosphorylated Cerebrospinal Fluid Tau in the Differential Diagnosis of Sporadic Creutzfeldt-Jakob Disease and Rapidly Progressive Alzheimer’s Disease

**DOI:** 10.3390/v14020276

**Published:** 2022-01-28

**Authors:** Peter Hermann, Philip Haller, Stefan Goebel, Timothy Bunck, Christian Schmidt, Jens Wiltfang, Inga Zerr

**Affiliations:** 1 Department of Neurology, National Reference Center for CJD Surveillance, University Medical Center Göttingen, 37075 Göttingen, Germany; philip.haller@stud.uni-goettingen.de (P.H.); stefan.goebel@med.uni-goettingen.de (S.G.); timothy.bunck@med.uni-goettingen.de (T.B.); Christian.Schmidt@medizin.uni-goettingen.de (C.S.); ingazerr@med.uni-goettingen.de (I.Z.); 2 Department of Psychiatry and Psychotherapy, University Medical Center Göttingen, 37075 Göttingen, Germany; jens.wiltfang@med.uni-goettingen.de; 3 German Center for Neurodegenerative Diseases (DZNE), 37075 Göttingen, Germany; 4 Neurosciences and Signaling Group, Department of Medical Sciences, Institute of Biomedicine (iBiMED), University of Aveiro, 3810-193 Aveiro, Portugal

**Keywords:** Creutzfeldt-Jakob disease, Alzheimer’s disease, rapidly-progressive dementia, biomarker, cerebrospinal fluid, tau, tau-ratio

## Abstract

Background: CSF total-tau (t-tau) became a standard cerebrospinal fluid biomarker in Alzheimer’s disease (AD). In parallel, extremely elevated levels were observed in Creutzfeldt-Jakob disease (CJD). Therefore, tau is also considered as an alternative CJD biomarker, potentially complicating the interpretation of results. We investigated CSF t-tau and the t-tau/phosphorylated tau181 ratio in the differential diagnosis of sCJD and rapidly-progressive AD (rpAD). In addition, high t-tau concentrations and associated tau-ratios were explored in an unselected laboratory cohort. Methods: Retrospective analyses included *n* = 310 patients with CJD (*n* = 205), non-rpAD (*n* = 65), and rpAD (*n* = 40). The diagnostic accuracies of biomarkers were calculated and compared. Differential diagnoses were evaluated in patients from a neurochemistry laboratory with CSF t-tau >1250 pg/mL (*n* = 199 out of 7036). Results: CSF t-tau showed an AUC of 0.942 in the discrimination of sCJD from AD and 0.918 in the discrimination from rpAD. The tau ratio showed significantly higher AUCs (*p* < 0.001) of 0.992 versus non-rpAD and 0.990 versus rpAD. In the neurochemistry cohort, prion diseases accounted for only 25% of very high CSF t-tau values. High tau-ratios were observed in CJD, but also in non-neurodegenerative diseases. Conclusions: CSF t-tau is a reliable biomarker for sCJD, but false positive results may occur, especially in rpAD and acute encephalopathies. The t-tau/p-tau ratio may improve the diagnostic accuracy in centers where specific biomarkers are not available.

## 1. Introduction

Prion diseases are caused by the propagation and aggregation of the misfolded prion protein scrapie (PrP^Sc^) in the brain [1]. Sporadic Creutzfeldt-Jakob disease (sCJD) is the most frequent form of human prion diseases, and accounts for around 90% of all cases, with an incidence of 1.5–2 per million person-years. It is clinically characterized by a rapidly-progressive encephalopathy, inevitably leading to death after a mean disease duration of 5–6 months [2]. The clinical phenotype is associated with distinct biochemical and morphological subtypes that are determined by the glycotype (type 1 or type 2) of the pathological prion protein (PrP^Sc^) and by the polymorphism at Codon 129 of the prion protein gene (PRNP) involving valine (V) and methionine (M) [3]. The most common subtype is MM1 and represents “classical” sCJD with rapidly progressive dementia, cerebellar syndrome, myoclonus, and a very short disease duration. Other subtypes may show predominant movement dysfunction (MV2 and VV2) in early stages or a prolonged disease duration (MV2, MM2, VV1). For many years, the diagnosis of sCJD has been based on criteria that included EEG, elevated CSF proteins 14-3-3, and MRI as biomarkers to support a probable clinical diagnosis [4,5]. Recently, the real-time quaking-induced conversion (RT-QuIC), which is able to detect PrP^Sc^ in CSF and other tissues with an excellent diagnostic accuracy, was included in revised consensus criteria [6]. Unfortunately, protein 14-3-3 and RT-QuIC analyses are usually only performed in specialized centers. In this context, CSF total Tau (t-tau), a microtubule-associated neuronal and glial protein [7], is considered as a valuable alternative biomarker with a good diagnostic accuracy [8] that might be improved by calculating a ratio with phosphorylated tau181 protein (t-tau/p-tau ratio) [9,10].

However, the interpretation of test results is complicated by a missing unified cut-off for the diagnosis of sCJD. In addition, elevated CSF t-tau is also widely employed in the diagnostic process for Alzheimer’s disease, indicating general neurodegeneration [11,12]. Although CSF t-tau values are much higher in sCJD than in AD, some studies reported that the discriminatory value versus clinically atypical AD may be reduced [13,14]. Further, highly elevated CSF t-tau concentrations were observed in patients with various non-neurodegenerative encephalopathies, such as acute ischemia, encephalitis, and after seizures [15].

The first aim of this study was to investigate the diagnostic accuracy of CSF t-tau in the differentiation of sCJD from AD and rapidly-progressive AD (rpAD), an AD subgroup that is defined by rapid cognitive decline [16], altered biomarker profiles [17], and potentially represents a disease entity with distinct beta-amyloid (abeta) strains [18]. In addition, we analyzed potential improvements of the diagnostic accuracy by calculating the t-tau/p-tau ratio. The second aim was to explore and describe the spectrum of differential diagnoses of patients with very high CSF t-tau values (above a pre-defined cut-off for sCJD) in a general neurochemistry laboratory cohort.

## 2. Materials and Methods

### 2.1. Study Cohorts

For this single-center study, a total number of *n* = 310 patients with sCJD (*n* = 205), non-rpAD (*n* = 65), and rpAD (*n* = 40) were included in the cohort for the evaluation of the diagnostic accuracy of t-tau, p-tau181, and the t-tau/p-tau ratio. Patients with sCJD were selected from a study of the National Reference Center for Transmissible Spongiform encephalopathies (NRZ-TSE) on epidemiology and biomarkers of prion diseases (ethical board number: 11/11/93). The selection criteria were the availability of the complete CSF tau biomarker dataset and neuropathological confirmation of definite CJD [4]. Patients with AD were selected from a prospective observational study on AD and rpAD (ethical board number: 6/9/08). The selection criteria were the availability of the complete CSF tau biomarker dataset, clinical diagnosis of probable AD [11], and sufficient follow-up information to differentiate between non-rpAD and rpAD. Further, concomitant CNS pathologies, especially clinically relevant cerebrovascular disease, inflammatory CNS diseases, and other neurodegenerative diseases, were ruled out as far as possible based on clinical syndrome and a complete diagnostic work-up, including CSF analyses and MRI. The rpAD group was defined by a loss of >5 points per year in each patient [16]. All analyzed CSF samples in both groups (AD and CJD) originated from lumbar punctures that had been performed during the diagnostic process (ante-mortem).

The second cohort included patients from the general neurochemistry laboratory of the Göttingen University Medical Center. Cases were selected on the base of the institution of treatment (only patients from the University Medical Center Göttingen were considered) and availability of CSF t-tau data. Between 2004 and 2019, CSF t-tau was analyzed in *n* = 7036 patients. Only patients above a previously defined CSF t-tau cut-off of >1250 pg/mL [19,20] were included for further evaluations (3%, *n* = 199). Diagnoses were evaluated based on information from the medical reports. All patients in both cohorts had given informed consent for the scientific evaluation of their anonymized data.

### 2.2. Biochemical Analyses

All CSF analyzes were performed in the neurochemistry lab of the Göttingen University Medical Center before conceptualization of this study during the diagnostic process; the technicians were blind to the final diagnosis. T-tau was measured using INNOTEST hTAU Ag ELISA Kit from Fujirebio. Tau phosphorylated at Thr181 was analyzed using INNOTEST ELISA kit PHOSPHO-TAU (181 P) from Fujirebio.

### 2.3. Statistical Methods

Multiple group comparisons were performed with univariate variance analyzes and Tukey HSD post hoc tests. The data was log transformed to achieve normalization. To calculate and demonstrate discriminatory values of biomarkers, Receiver Operator Characteristics (ROC) were carried out. The area under the ROC-Curve (AUC) with according 95% intervals (95%CI) was considered as measure for the diagnostic accuracy. Optimal cut-offs were calculated using the Youden-Index. DeLong’s-Tests [21] were performed to investigate the differences between ROC-curves of the biomarkers.

In *n* = 7 sCJD cases, the test-ELISA kit had produced a t-tau value of >2200 pg/mL, and further dilution to determine an exact (higher) value was not performed. A value of 2200 pg/mL was assumed in these cases and used in statistical calculations to avoid favoring the hypothesis of higher values in sCJD. Statistical analyses were performed with Jamovi^®^ in R^®^ and SPSS.

## 3. Results

### 3.1. Demographic Data and Biomarker Values in the Study Cohort

The two major diagnostic groups showed similar age characteristic with a median of 70 (IQR 16.5) in AD and 68 (IQR 14.5) in sCJD years patients. Some sCJD subtypes showed younger age medians, especially MV2 (61 years, IQR 12) and VV1 (51, IQR 36.0 years). Regarding sex distribution, 56% of patients in the AD group and 46% of patients in the sCJD group were female. Interestingly, the sex distribution in the rpAD group differed substantially with 68% of the patients being female (Table 1).

The proteins 14-3-3 showed an intermediate (or “weak positive”) Western Blot signal in the CSF of four AD patients (7%), all of them belonging to the rpAD subgroup. CSF 14-3-3 was positive or intermediate in *n* = 188 patients from the sCJD group (92%). The median t-tau concentration in sCJD patients was 4840 pg/mL (IQR: 6882.5) and 546 pg/mL (IQR: 511) in AD patients. T-tau concentrations in sCJD and AD subgroups can be found in Table 1.

**Figure 1:**
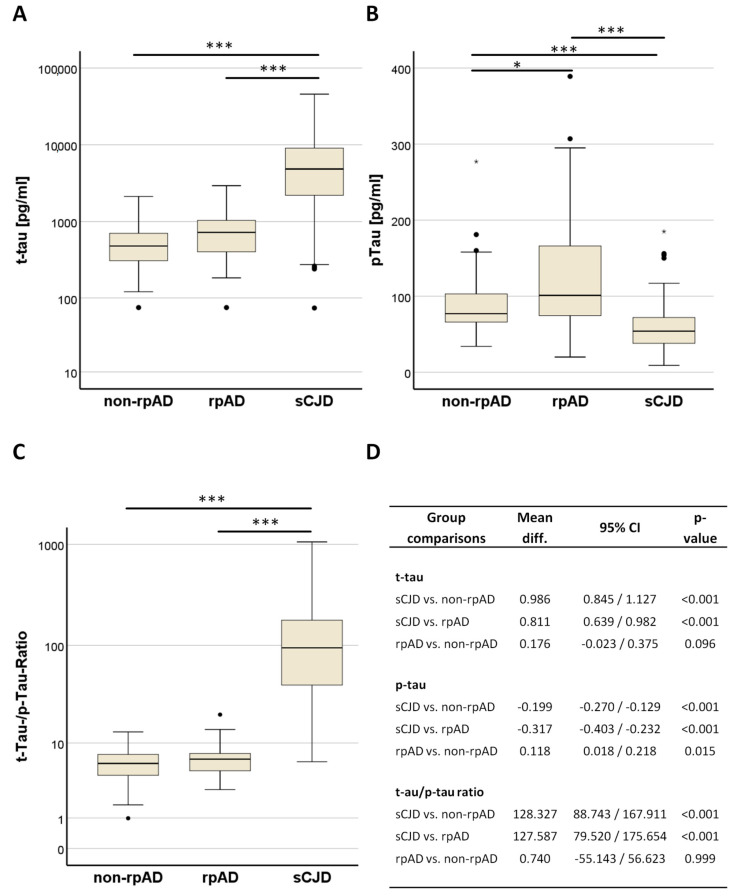
T-tau and p-tau181 levels in sCJD, classical (non-rp) AD, and rpAD. (**A**) Box plot of cerebrospinal fluid total-Tau (t-au) levels in non-rapidly-progressive Alzheimer’s disease (non-rpAD) patients, rapidly-progressive AD (rpAD), and sporadic Creutzfeldt-Jakob disease (sCJD) patients. (**B**) Box plot of cerebrospinal fluid phosphorylated Tau181 protein (p-tau) levels in non-rpAD, rpAD, and sCJD patients. (**C**) Box plot of t-tau/p-tau ratio values in non-rpAD, rpAD, and sCJD patients. (**D**) Mean difference, 95% confidence intervals (CI), and according *p*-values from univariate variance analyzes and post hoc Tukey HSD tests for multiple comparisons between log-transferred biomarker levels and ratio values. Bars above box plots (**A**–**C**) indicate significance levels from the comparison model. * *p* < 0.05; *** *p* < 0.001. Black dots above and below boxes indicate outliers. To improve readability, logarithmic scaling was chosen for x-axes in (**A**,**C**).

In the multiple group comparison, t-tau was significantly higher in sCJD than in non-rpAD (*p* < 0.001) and rpAD (*p* < 0.001). RpAD cases showed higher t-tau concentrations (median 724 pg/mL, IQR: 633) than non-rpAD cases (median 480 pg/mL, IQR: 404.5) without statistical significance (*p* = 0.096) (Figure 1A,D). In contrast, p-tau181 was lower in sCJD (median 54 pg/mL, IQR: 34) than in non-rpAD (median 77 pg/mL, IQR: 38), and rpAD (median 101 pg/mL, IQR: 91.5). Interestingly, p-tau181 was not only lower in sCJD compared to non-rpAD and rpAD (each *p* < 0.001), but also significantly lower in non-rpAD than in rpAD (*p* = 0.015) (Figure 1B,D). The t-tau/p-tau ratio showed a pattern very similar to t-tau, with significantly higher values in sCJD than in non-rpAD and in rpAD (each *p* < 0.001). Here, the difference between non-rpAD and rpAD was marginal (*p* = 0.999) (Figure 1C,D).

**Table 1:** Demographic and biomarker data of the study cohort.

Group	*n*	Sex (Female/ Male)	Age (Median, IQR)	14-3-3 (neg./Interm./pos.)	t-tau [pg/mL] Median (IQR)	p-tau181 [pg/mL] Median (IQR)	t-tau/p-Tau Ratio Median (IQR)
AD	105	59/46	70 (16.5)	52/4/0 *	546 (511)	82 (49)	6.2 (3.0)
non-rpAD	65	32/33	70 (17.0)	33/0/0 *	480 (404.5)	77 (38)	5.9 (3.2)
rpAD	40	27/13	71 (14.0)	19/4/0 *	724 (633)	101 (91.5)	6.6 (2.8)
sCJD	205	95/110	68 (14.5)	165/23/16 **	4840 (6882.5)	54 (34)	94.5 (138.8)
MM/MV1	63	31/32	69 (12.0)	60/2/1	7212 (6875)	43 (32)	165.0 (160.8)
MV2	12	6/6	61 (12.0)	4/0/8	2050.5 (2886)	53.5 (21.5)	30.9 (36.6)
VV2	17	9/8	65 (17.0)	16/0/1	5993 (7379.5)	55 (25.5)	115.3 (120.6)
MM2C	7	3/4	70 (10.0)	6/1/0	3093 (3935)	54 (46)	70.3 (65.7)
VV1	4	1/3	51 (36.0)	4/0/0	4607.5 (7587)	52.5 (49)	89.1 (52.9)
MM1+2	3 ***	0/3	73 (NA)	1/2/0	1940/1625/2571	50/26/37	38.8/62.5/ 69.5

* Retrospective data on 14-3-3 is incomplete, analyses were performed with Western Blot, which is not performed at the NRZ-TSE anymore, data on 14-3-3 ELISA was not available; ** results are partially from Western Blot (*n* = 183) and from ELISA (*n* = 21) analyses; *** MM1+2 CJD subtype was present in three cases, no biomarker median but each value is shown; spAD: slowly progressive Alzheimer’s disease; rpAD: rapidly progressive Alzheimer’s disease; sCJD: sporadic Creutzfeldt-Jakob disease; MM: methionine homozygosity at Codon 129 *PRNP*; MV: methionine/valin heterozygosity; VV: valin homozygosity.

### 3.2. Diagnostic Accuracy of CSF t-tau, p-tau181, and the t-/p-tau Ratio in the Study Cohort

**Figure 2:**
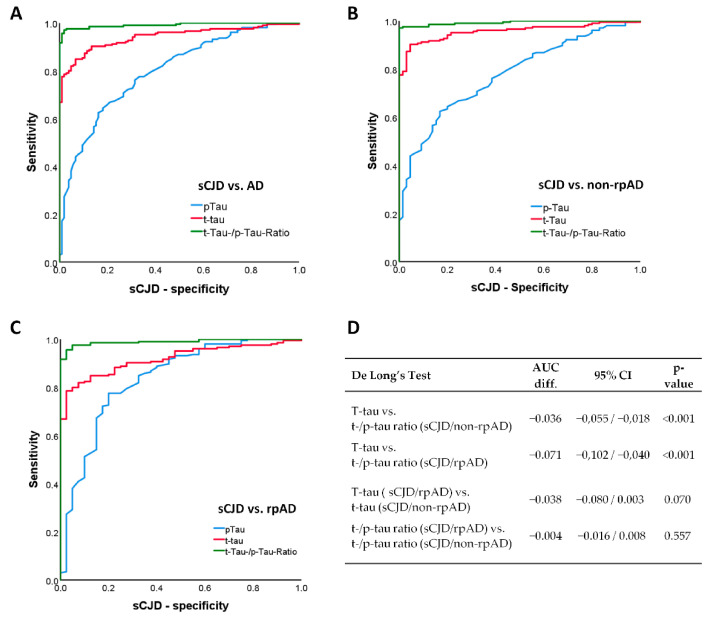
2ROC-Analyses of t-tau, p-tau181, and their ratio in the discrimination of AD and rpAD from sCJD. (**A**) Receiver operating characteristics (ROC) in the discrimination of sporadic Creutzfeldt-Jacob disease (sCJD) from Alzheimer’s disease AD, displaying total cerebrospinal fluid Tau (t-tau, red line), phosphorylated cerebrospinal fluid Tau (p-tau181, blue line), and the t-tau/p-tau ratio (green). (**B**) Receiver operating characteristics (ROC) in the discrimination of sCJD from non-rapidly-progressive Alzheimer’s disease (non-rpAD), displaying t-tau (red line), p-tau181 (blue line), and the t-tau/p-tau ratio (t-tau/p-tau ratio) (green). (**C**) Receiver operating characteristics (ROC) in the discrimination of sCJD from rapidly-progressive Alzheimer’s disease (rpAD), displaying t-tau (red line), p-tau181 (blue line), and the t-tau/p-tau ratio (green). (**D**) Comparison and indication of significant differences with 95% confidence intervals (CI) between Areas Under the Curve (AUC) from ROC analyzes.

CSF t-tau discriminated sCJD from the whole AD group with an AUC of 0.942 (95%CI: 0.917–0.967) at an optimal cut-off of 1583 pg/mL. At this concentration, the sensitivity was 85% and the specificity 93%. The AUCs in the differentiation of sCJD and non-rpAD (0.957, 95%CI: 0.934–0.979) and rpAD (0.918, 0.884–0.953) were also very high, but optimal cut-offs differed to a rather great extent between non-rpAD (>990 pg/mL) and rpAD (>2045 pg/mL). CSF p-tau181 showed moderate to good diagnostic accuracy with AUCs of 0.799 (95%CI: 0.748–0.849) vs. all AD, 0.776 (0.718–0.835) vs. non-rpAD, and 0.835 (95%CI: 0.761–0.090) vs. rpAD. The optimal cut-offs were <62 pg/mL vs. AD and non-rpAD, and <72 pg/mL vs rpAD. The t-au/p-tau ratio showed an excellent diagnostic accuracy in the discrimination of sCJD and AD as well as all subgroups (each AUC ≥ 0.990) at similar cut-off values of >13 vs. AD and the non-rpAD subgroup and >14 vs. rpAD patients. Please see Table 2 for a summary of the data and according ROC curves in Figure 2A–C.

In a second step, we compared the obtained AUCs. Here, the t-tau/p-tau ratio performed significantly better than CSF t-tau alone in the discrimination of sCJD from non-rpAD (AUC difference: −0.036, 95%CI: −0.055 to −0.018, *p* < 0.001) and from rpAD (AUC difference: −0.071, 95%CI:−0.102 to −0.040, *p* < 0.001). In addition, we compared the AUCs of CSF t-tau in the discrimination of sCJD from non-rpAD than and from rpAD. Although the AUC vs. rpAD was lower than vs. non-rpAD (AUC difference: −0.038, 95%CI: −0.080 to −0.003), the difference did not pass the significance threshold (*p* = 0.070). Regarding the t-tau/p-tau ratio, the AUC difference between non-rpAD and rpAD was marginal (AUC difference: −0.004, 95%CI: −0.016 to 0.008, *p* = 0.557). Please see Figure 2D for a summary of the data.

### 3.3. Diagnostic Accuracy in sCJD Subtypes

Information on disease subtype, including PrPSc glycotype and Codon129 PRNP genotype, were available in a subset of *n* = 106 patients. Regarding CSF t-tau, MM2C and MV2K sCJD showed lower concentrations than other subtypes (Table 1), in line with finding in the literature [22]. However, we did not statistically compare all biomarker values over all six observed groups because case numbers, especially in MM2C, VV1, and mixed types, were very low. For the same reason, we concentrated evaluations of the diagnostic accuracy on the three most common subtypes: MM/MV1, VV2, and MV2K. CSF t-tau showed the best accuracy in the differentiation of MM/MV1 cases from AD cases at a cut-off of >2045 pg/mL (0.977, 95%CI: 0.944–1). In VV2 cases, CSF t-tau also showed high AUCs of >0.900 vs. all AD types, but in MV2K cases, especially vs. rpAD, the AUC was lower (0.792, 95%CI: 0.646–0.937). This was not the case for the t-tau/p-tau ratio, which showed AUCs > 0.985 in all sCJD subtypes vs. all AD types (Table 3).

### 3.4. Exploration of High CSF t-Tau Values in a General Neurochemistry Laboratory

**Figure 3:**
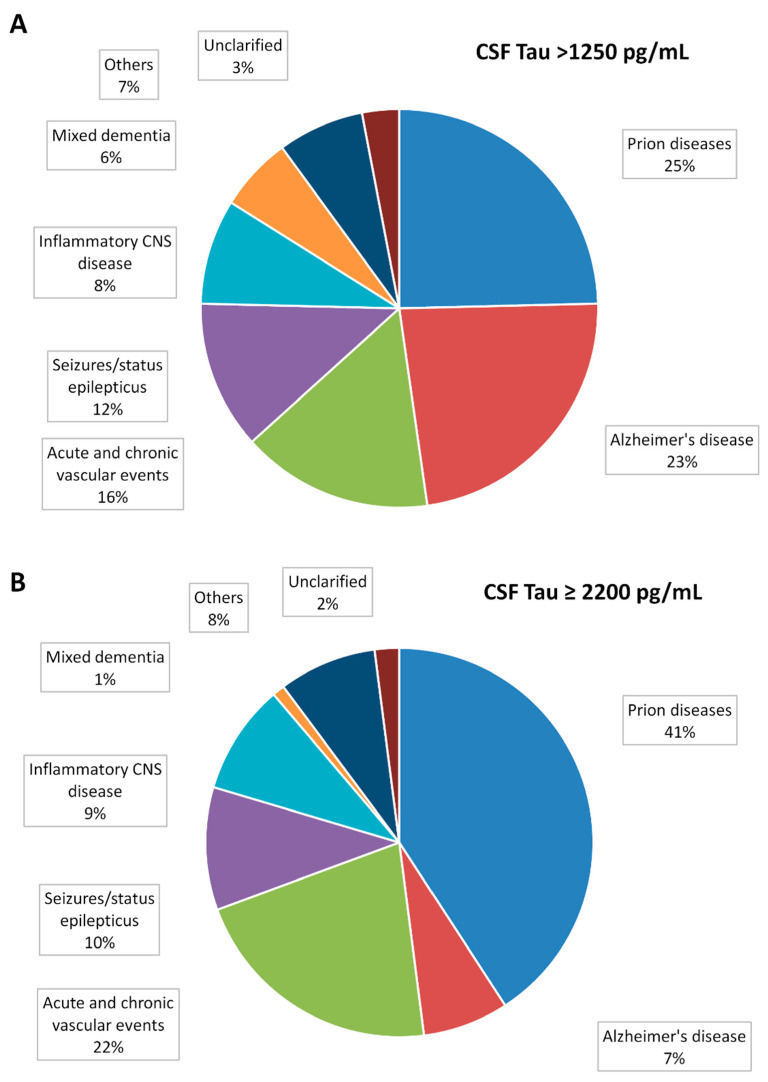
Differential diagnoses of high CSF t-Tau concentrations (**A**) Pie chart: Differential diagnoses of all cases with cerebrospinal fluid total-Tau >1250 pg/mL analyzed in the neurochemistry lab of the Göttingen University Medical Center between 2010 and 2019 (total *n* = 199). Prion diseases were present in *n* = 49 (25%), Alzheimer’s disease in *n* = 46 (23%), acute and chronic vascular encephalopathy in *n* = 31 (16%), seizures in *n* = 24 (12%), inflammatory CNS disease in *n* = 17 (9%), and mixed neurodegenerative dementia in *n* = 12 (6%) cases. Other conditions (normal pressure hydrocephalus, other neurodegenerative dementia, hypoxic brain damage, toxic-metabolic encephalopathy, MELAS, and CNS Neoplasia) were present in *n* = 14 (7%) cases, *n* = 6 (3%) cases remained unclassified. (**B**) Pie chart: Differential diagnoses of all cases with cerebrospinal fluid total-Tau 2200 pg/mL (*n* = 98). Prion diseases were present in *n* = 40 (41%), Alzheimer’s disease in *n* = 7 (7%), acute and chronic vascular encephalopathy in *n* = 21 (21%), seizures in *n* = 10 (10%), and inflammatory CNS disease in *n* = 9 (9%). Other conditions (normal pressure hydrocephalus, other neurodegenerative dementia, hypoxic brain damage, toxic-metabolic encephalopathy, and MELAS) were present in *n* = 11 (11%) patients.

The second part of the study evaluated differential diagnoses in patients with high CSF t-tau values between 2004 and 2019 that had been referred to the Göttingen University Medical Center and analyzed in the institutional neurochemistry laboratory. Out of *n* = 7036, we identified *n* = 199 patients with CSF > 1250 pg/mL (3%). Their diagnoses and the associated numbers of cases are displayed in Figure 3A. About 25% of these patients were diagnosed with prion diseases. The second largest group were patients with AD (23%), followed by acute (stroke) and chronic vascular encephalopathy (16%), seizures (12%), inflammatory CNS disease in (9%), and mixed neurodegenerative dementia in *n* = 12 (6%). Other conditions were present in 7% of the cases and 3% cases unclassified according to available clinical data. In cases with CSF t-tau ≥ 2200 pg/mL, prion diseases accounted for 41% of the cases, whereas the frequency of AD (7%) and MD (1%) was substantially lower (Figure 3B).

**Figure 4:**
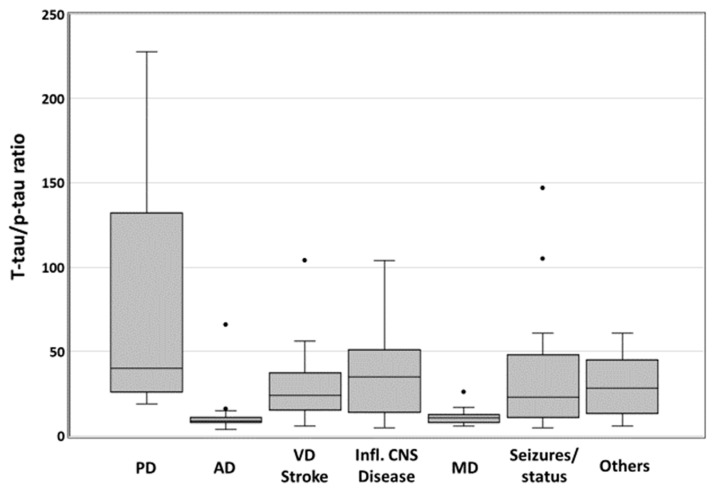
T-tau/p-tau ratio in differential diagnoses of CSF t-tau values >1250 pg/mL. Boxplot of t-tau/p-tau ratios (*n* = 170) in patients with CSF t-tau >1250 pg/mL. PD: Prion disease (*n* = 34, median: 40.56), AD: Alzheimer’s disease (*n* = 46, median: 9.80), VD/Stroke: acute and chronic vascular encephalopathy (*n* = 28, median: 24.69), inflammatory CNS diseases (*n* = 15, median: 35.06), MD: mixed AD and vascular dementia (*n* = 12, median: 10.85), seizures and status epilepticus (*n* = 19, median 23.44), and others (*n* = 16, median: 29.19). Black dots above boxes indicate outliers.

Regarding the t-tau/p-tau ratio, data was available in *n* = 170 cases. As this cohort was preselected by a minimum CSF t-tau of 1250 pg/mL and many t-tau concentrations above the maximum laboratory standard of 2200 pg/mL were present, we did not statistically analyze the data with group comparisons and ROC curves. Instead, we describe the distribution of t-tau/p-tau medians over the diagnostic groups. Here, prion diseases showed the highest medians, (40.56) and AD the lowest (9.80) medians. Non-neurodegenerative conditions such as vascular events (24.69), seizures (23.44), and inflammatory CNS diseases (35.06) showed t-tau/p-tau values higher than AD and lower than prion diseases (Figure 4).

## 4. Discussion

The results of our investigation validate the good diagnostic accuracy of CSF t-tau in the differential diagnosis of sCJD in the context of AD and rpAD. However, the optimal cut-off (>1583 pg/mL versus AD) was higher than what can be found in the literature. In previous publications, CSF t-tau cut-offs between >1072 pg [23] and >1400 pg/mL [24] were identified using the heterogeneous control groups of non-CJD neurologic diseases or rapidly-progressive dementias of different etiologies [8,9,19,25,26]. Studies that used AD patients as controls showed differing results. AUCs varied between 0.93 with a cut-off of >2131 pg/mL [27] and 0.78 with a cut-off of >1200 pg/mL [14]. Another study showed a relatively high AUC of 0.92 at a rather low cut-off >1128 pg/mL [13]. This study used only “typical AD” cases for the evaluation, and the results were similar to those from our subgroup analysis of non-rpAD (AUC 0.96, cut-off: >990 pg/mL) cases. Lower diagnostic accuracies were reported when atypical forms of AD were included or focused [13,14]. In our study, we defined rpAD by pre-existing criteria [16,28] as a distinct AD subgroup in biomarker analyses. We partially validated previous observations that investigated so-called atypical AD and showed that the AUC of CSF t-tau for the discrimination of sCJD was lower vs. rpAD than vs. non-rpAD (AUC difference: −0.038). However, the *p*-value from DeLong’s test (0.070) stayed above the pre-defined threshold for statistical significance. In addition, the optimal cut-off to discriminate the rpAD group (>2045 pg/mL) was substantially higher. It was shown before that rpAD may be characterized by a distinct biomarker profile [17] and that a faster disease progression goes along with higher values of biomarkers of neuronal damage [29]. The latter has also been shown for sCJD [30]. Nonetheless, the difference between the diagnostic accuracy in non-AD and rpAD in our study was not as clear as the difference between typical and atypical AD in the aforementioned studies. The potential reason may be the selection and definition of the AD group. Whereas many studies investigated the diagnostic accuracy of CSF tau using patients with AD that had initially been suspected as sCJD, we analyzed CSF from rpAD patients that were part of an independent study on AD, reflecting the spectrum of AD in a non-specialized center.

We could not identify a significant elevation of CSF t-tau in rpAD compared to non-rpAD (*p* = 0.096) patients but we observed significantly higher p-tau181 values in the rpAD group (*p* = 0.015). This comparison was not a main objective of the study, but the results are important and match with findings in atypical AD [13,14]. However, unlike other studies [17], we could not find significant differences between the t-tau/p-tau ratios in rpAD and non-rpAD patients (*p* = 0.999).

The t-tau/p-tau ratio is a major improvement to the use of CSF t-tau in the differential diagnosis of sCJD, which was demonstrated by several studies [9,10,26,31,32,33]. Here, we validate those findings and were able to show significantly higher AUCs vs. non-rpAD as well as rpAD (*p* < 0.001). Most important, there is only a marginal and non-significant difference between AUCs for the discrimination of sCJD from non-rpAD and from rpAD (*p* = 0.557). This indicates that the t-tau/p-tau ratio may be robust and less susceptible to show false positive results in AD patients with very high CSF t-tau values. In addition, we could show that the good diagnostic performance remains constant not only over AD groups, but also regarding different sCJD subtypes. Whereas the AUCs of t-tau were rather low (0.792) in the discrimination of rpAD and MV2 (0.792) and very high in MM/MV1 versus non-rpAD (0.979), the AUCs of the t-tau/ p-tau ratio showed values > 0.980 in all ROC analyzes (Table 3).

Very high CSF t-tau values may not only occur in sCJD and a proportion of AD patients. It was shown that CSF t-tau is not markedly elevated in other neurodegenerative dementias [34], but as a general marker of neuro-axonal damage, very high concentrations were observed after cerebral ischemia [35], hemorrhage, seizures [36], as well as in encephalitis and other conditions [15]. In the cohort from the general neurochemistry laboratory, only about half of the patients with very high CSF-tau values (>1250 pg/mL) were diagnosed with prion diseases (25%), AD (23%), or other neurodegenerative diseases (mixed or single pathologies: 7%). Studies with similar approaches have reported that the majority of patients with CSF t-tau >1000 pg/mL [37] and >1200 pg/mL [38] were diagnosed with AD (73% and 51%, respectively), followed by CJD. A potential reason for this discrepancy may the slightly higher cut-off used in this study and the fact that the German NRZ-TSE is located at the Göttingen University. The frequency of patients with prion diseases is higher in this institution due to second opinion referrals.

Unlike in AD and other secondary tauopathies [39], elevated t-tau may not go along with elevated p-tau181 in non-neurodegenerative encephalopathies [35]. This may potentially be a reason for a lower diagnostic accuracy in some CJD mimics. In our exploratory evaluation of patients with CSF t-tau >1250 pg/mL, values of the t-tau/p-tau ratio in AD patients were apparently lower than in inflammatory and vascular encephalopathies. These conditions showed a huge overlap with prion diseases (Figure 3B). This is of high importance, because inflammatory and neurovascular diseases belong to the most common differential diagnoses of sCJD and rapidly-progressive dementia [40].

The strengths of this study include the use of a well-characterized “real-life” AD control group with a rather impartial criterion for the definition of rpAD [16], the consideration of different subgroups in AD as well as in sCJD, and a high case number in the sCJD group. Focusing on only one disease as differential diagnosis and on one CSF biomarker category (14-3-3 data was not available for the AD cohort) was also a limitation of the study. Unfortunately, the retrospective study design did not allow comparative evaluations with ELISA 14-3-3, beta-amyloid 1–42, or recent biomarker candidates for CJD such as neurofilament light chain, alpha-synuclein, or soluble triggering receptor expressed on myeloid cells 2 (TREM2). Future investigations should also consider potential improvements of the diagnostic accuracy by combinations of different biomarkers, as well as consideration of clinical (e.g., disease stage) and demographic factors [41]. The lack of valid comparative data on the t-tau/p-tau ratio in the laboratory cohort is another important limitation. It emphasizes the need to take non-neurodegenerative dementia etiologies into account when performing future evaluations of the diagnostic accuracy of the t-tau/p-tau ratio and other biomarkers for prion diseases.

The current focus of biomarker research in CJD, AD, and other dementias lies on blood-based analyses, and the utility of plasma t-tau for the differential diagnosis of sCJD has already been validated [42]. In this context, an investigation of potential improvements through the application of a plasma t-tau/p-tau ratio as well as other promising tau markers such as non-phosphorylated tau [43] and p-tau217 [44] should be considered in future research.

## 5. Conclusions

CSF t-tau is a valuable alternative biomarker for sCJD when specific tests like RT-QuIC are not available. However, very high t-tau values may occur in other diseases, especially in rpAD and in the non-neurodegenerative etiologies of rapidly-progressive dementia. The t-tau/p-tau ratio is able to improve the diagnostic accuracy for the discrimination of sCJD from AD and rpAD significantly, but its utility in the context of ischemic and inflammatory encephalopathies has to be explored further. Although we and others reported excellent accuracies of CSF t-tau and the t-tau/p-tau ratio, predictive values of these biomarkers are mainly determined by the extremely low prevalence of sCJD. Thus, CSF t-tau should not be used as a general screening test for sCJD, and incidental findings of very high concentrations in the diagnostic process of a suspected AD, as well as non-neurodegenerative encephalopathies, have to be interpreted with caution.

## Figures and Tables

**Table 2:** AUCs and best cut-offs of t-tau, p-tau181, and the p-tau/t-tau ratio.

Groups and Biomarkers	Cutoff [pg/mL]	Sensitivity [%]	Specificity [%]	AUC	95%CI
**t-tau**					
sCJK vs. AD	>1583	85	93	0.942	0.917–0.967
sCJK vs. rpAD	>2045	79	98	0.918	0.884–0.953
sCJK vs. non-rpAD	>990	90	95	0.957	0.934–0.979
**p-Tau181**					
sCJK vs. AD	<62	84	62	0.799	0.748–0.849
sCJK vs. rpAD	<74	80	78	0.835	0.761–0.090
sCJK vs. non-rpAD	<62	83	62	0.776	0.718–0.835
**t-/p-Tau-Ratio**					
sCJK vs. AD	>13	97	98	0.992	0.984–1
sCJK vs. rpAD	>14	96	98	0.990	0.980–0.999
sCJK vs. non-rpAD	>13	97	100	0.993	0.086–1

spAD: slowly progressive Alzheimer’s disease; rpAD: rapidly progressive Alzheimer’s disease; sCJD: sporadic Creutzfeldt-Jakob disease; ideal cut-off, calculated by the youden ratio; AUC: area under the curve from receiver operator characteristics; CI: confidential interval. Bold subheadings indicate the biomarker used for receiver operator characteristics.

**Table 3:** CSF Tau in the discrimination of AD and sCJD subtype.

Groups and Biomarkers	Cutoff [pg/mL]	Sensitivity [%]	Specificity [%]	AUC	95%CI
**t-Tau**					
MM1/MV1 vs. AD	>2045	94	98	0.977	0.944–1
MM1/MV1 vs. rpAD	>2045	94	98	0.972	0.937–1
MM1/MV1 vs. non-rpAD	>1667	97	97	0.979	0.948–1
VV2 vs. AD	>2730	88	99	0.916	0.805–1
VV2 vs. rpAD	>2730	88	98	0.904	0.780–1
VV2 vs. non-rpAD	>2730	88	100	0.923	0.820–1
MV2 vs. AD	>773	92	70	0.868	0.770–0.967
MV2 vs. rpAD	>2022	50	98	0.792	0.646–0.937
MV2 vs non-rpAD	>773	92	80	0.915	0.834–0.996
**p-Tau181**					
MM1/MV1 vs. AD	<66	79	80	0.862	0.805–0.919
MM1/MV1 vs. rpAD	<70	83	84	0.882	0.812–0.953
MM1/MV1 vs. non-rpAD	<62	83	73	0.850	0.784–0.915
VV2 vs. AD	<73	69	88	0.833	0.741–0.926
VV2 vs. rpAD	<74	80	88	0.865	0.772–0.959
VV2 vs. non-rpAD	<65	77	76	0.814	0.703–0.927
MV2 vs. AD	<62	84	75	0.803	0.667–0.938
MV2 vs. rpAD	<74	80	83	0.831	0.708–0.955
MV2 vs. non-rpAD	<62	83	75	0.785	0.628–0.942
**t-/p-Tau-Ratio**					
MM1/MV1 vs. AD	>26	98	100	0.992	0.976–1
MM1/MV1 vs. rpAD	>26	98	100	0.991	0.973–1
MM1/MV1 vs. non-rpAD	>26	98	100	0.993	0.978–1
VV2 vs. AD	>39	94	100	0.984	0.954–1
VV2 vs. rpAD	>39	94	100	0.981	0.943–1
VV2 vs. non-rpAD	>39	94	100	0.986	0.959–1
MV2 vs. AD	>14	100	99	0.998	0.992–1
MV2 vs. rpAD	>14	100	98	0.994	0.979–1
MV2 vs. non-rpAD	>14	100	100	1	-

spAD: slowly progressive Alzheimer’s disease; rpAD: rapidly progressive Alzheimer’s disease; sCJD: sporadic Creutzfeldt-Jakob disease; MM: methionine homozygosity at Codon 129 PRNP; MV: methionine/valin heterozygosity; VV: valin homozygosity; AUC: area under the curve from receiver operator characteristics; CI: confidential interval. Bold subheadings indicate the biomarker used for receiver operator characteristics.

## Data Availability

The data presented in this study are available on request from the corresponding author.
